# Transcriptome Dynamics of the Inflorescence in Reciprocally Formed Allopolyploid *Tragopogon miscellus* (Asteraceae)

**DOI:** 10.3389/fgene.2020.00888

**Published:** 2020-08-06

**Authors:** Shengchen Shan, J. Lucas Boatwright, Xiaoxian Liu, Andre S. Chanderbali, Chaonan Fu, Pamela S. Soltis, Douglas E. Soltis

**Affiliations:** ^1^Plant Molecular and Cellular Biology Program, University of Florida, Gainesville, FL, United States; ^2^Florida Museum of Natural History, University of Florida, Gainesville, FL, United States; ^3^Advanced Plant Technology Program, Clemson University, Clemson, SC, United States; ^4^Department of Biology, University of Florida, Gainesville, FL, United States; ^5^Environmental Genomics and Systems Biology (EGSB), Biosciences Area, Lawrence Berkeley National Laboratory, Berkeley, CA, United States; ^6^Key Laboratory for Plant Diversity and Biogeography of East Asia, Kunming Institute of Botany, Chinese Academy of Sciences, Kunming, China; ^7^Biodiversity Institute, University of Florida, Gainesville, FL, United States; ^8^Genetics Institute, University of Florida, Gainesville, FL, United States

**Keywords:** homeolog, inflorescence, non-additive expression, polyploidy, reciprocal formation, *Tragopogon*, transcriptome

## Abstract

Polyploidy is an important evolutionary mechanism and is prevalent among land plants. Most polyploid species examined have multiple origins, which provide genetic diversity and may enhance the success of polyploids. In some polyploids, recurrent origins can result from reciprocal crosses between the same diploid progenitors. Although great progress has been made in understanding the genetic consequences of polyploidy, the genetic implications of reciprocal polyploidization remain poorly understood, especially in natural polyploids. *Tragopogon* (Asteraceae) has become an evolutionary model system for studies of recent and recurrent polyploidy. Allotetraploid *T. miscellus* has formed reciprocally in nature with resultant distinctive floral and inflorescence morphologies (i.e., short- vs. long-liguled forms). In this study, we performed comparative inflorescence transcriptome analyses of reciprocally formed *T. miscellus* and its diploid parents, *T. dubius* and *T. pratensis*. In both forms of *T. miscellus*, homeolog expression of ∼70% of the loci showed vertical transmission of the parental expression patterns (i.e., parental legacy), and ∼20% of the loci showed biased homeolog expression, which was unbalanced toward *T. pratensis*. However, 17.9% of orthologous pairs showed different homeolog expression patterns between the two forms of *T. miscellus*. No clear effect of cytonuclear interaction on biased expression of the maternal homeolog was found. In terms of the total expression level of the homeologs studied, 22.6% and 16.2% of the loci displayed non-additive expression in short- and long-liguled *T. miscellus*, respectively. Unbalanced expression level dominance toward *T. pratensis* was observed in both forms of *T. miscellus*. Significantly, genes annotated as being involved in pectin catabolic processes were highly expressed in long-liguled *T. miscellus* relative to the short-liguled form, and the majority of these differentially expressed genes were transgressively down-regulated in short-liguled *T. miscellus*. Given the known role of these genes in cell expansion, they may play a role in the differing floral and inflorescence morphologies of the two forms. In summary, the overall inflorescence transcriptome profiles are highly similar between reciprocal origins of *T. miscellus*. However, the dynamic homeolog-specific expression and non-additive expression patterns observed in *T. miscellus* emphasize the importance of reciprocal origins in promoting the genetic diversity of polyploids.

## Introduction

Polyploidy, also known as whole-genome duplication (WGD), is a major evolutionary force in all eukaryotes (Otto, 2007; Soltis et al., 2014, 2016; Wendel, 2015; Van de Peer et al., 2017). WGD events are particularly prevalent and important in land plants, especially ferns and flowering plants (Leebens-Mack et al., 2019). Many ancient WGD events occur near the origin of several large angiosperm clades and are accompanied by the evolution of novel traits (Soltis et al., 2009; Soltis and Soltis, 2016), adaptation to dramatic environmental changes (Wu et al., 2019), and rapid niche differentiation (Baniaga et al., 2020).

Two types of polyploids are generally recognized: allopolyploids are formed by hybridization and chromosome doubling between two species, whereas autopolyploids are derived from genome duplication within a species (Grant, 1981). In both allopolyploids and autopolyploids, recent studies have illustrated the dynamic nature of polyploid genome evolution at multiple levels, including: chromosomal variation, gene loss, homeolog (duplicated gene copies following allopolyploidy) expression bias (see Box 1 for all terminologies used in this article), non-additive gene expression (including expression level dominance and transgressive expression; Box 1), transposable element activation, and epigenetic changes (reviewed in Osborn et al., 2003; Doyle et al., 2008; Jackson and Chen, 2010; Mayfield et al., 2011; De Smet and Van de Peer, 2012; Madlung and Wendel, 2013; Buggs et al., 2014; Soltis et al., 2014; Yoo et al., 2014; Spoelhof et al., 2017; Wendel et al., 2018; Doyle and Coate, 2019). In addition, homeolog expression bias and expression level dominance can be either balanced or unbalanced (Box 1; Grover et al., 2012).

**Box 1. Terminology of the gene expression profiles in allopolyploids used in this article.**

**Table:** 

**Type of analysis**	**Terminology**	**Definition**
Homeolog-specific expression analysis	Homeolog expression bias	Unequal expression levels of the two parental homeologs of a gene in the allopolyploid.
	Balanced homeolog expression bias	When multiple genes are examined, equivalent number of genes display homeolog expression bias toward each parent.
	Unbalanced homeolog expression bias	When multiple genes are examined, more genes show homeolog expression bias toward one parental genome than the other.
	Parental legacy	When comparing the allopolyploid and its diploid progenitors, the expression patterns of the diploid parents are vertically transmitted to the allopolyploid (Figure 2A, categories 1–3).
	Absence of homeolog expression bias	The diploid parents are differentially expressed at a given locus, but homeolog expression of that gene is unbiased in the allopolyploid (Figure 2A, categories 4 and 5).
	Novel homeolog expression	Homeolog expression of a gene in the allopolyploid is biased toward the diploid parent that displays an equal or lower expression level than the other diploid parent at that locus (Figure 2A, categories 6–9).
Total gene expression analysis (combining the expression levels of both homeologs of a gene)	Expression level dominance	One type of non-additive gene expression in which the expression level of a gene in the allopolyploid is equivalent to only one of the diploid progenitors.
	Balanced expression level dominance	When multiple genes are examined, equivalent number of genes display expression level dominance toward each parent.
	Unbalanced expression level dominance	When multiple genes are examined, more genes show expression level dominance toward one parental genome than the other.
	Transgressive expression	A second type of non-additive gene expression in which the gene expression level in the allopolyploid is higher (transgressive up-regulation) or lower (transgressive down-regulation) than in both diploid parents.

Significantly, most polyploids that have been investigated genetically at the population level have formed repeatedly (Soltis and Soltis, 1999; Soltis et al., 2004). That is, the same polyploid species has formed multiple times from genetically different diploid individuals. Because of the genetic diversity of the parents, independent assortment and recombination within polyploids, and subsequent gene flow among polyploid populations, multiple origins may have an important influence on the genetic diversity of polyploids.

As a special case of multiple origins, reciprocal origins of allopolyploids having similar nuclear genomes but distinct cytoplasmic genomes have been documented in various species, including *Aegilops* spp. (Meimberg et al., 2009), *Androsace brigantiaca* (Dixon et al., 2009), *Asplenium* spp. (Perrie et al., 2010; Sessa et al., 2018), *Brassica napus* (Song and Osborn, 1992), *Platanthera huronensis* (Wallace, 2003), *Polypodium hesperium* (Sigel et al., 2014), *Senecio* spp. (Kadereit et al., 2006), and *Tragopogon miscellus* (Ownbey and McCollum, 1953; Soltis and Soltis, 1989). Many reciprocally formed polyploids are divergent in morphology and/or geographic distribution. However, reciprocal polyploidization remains poorly studied, and its genetic impact in natural polyploid populations is still largely unknown.

**FIGURE 1:**
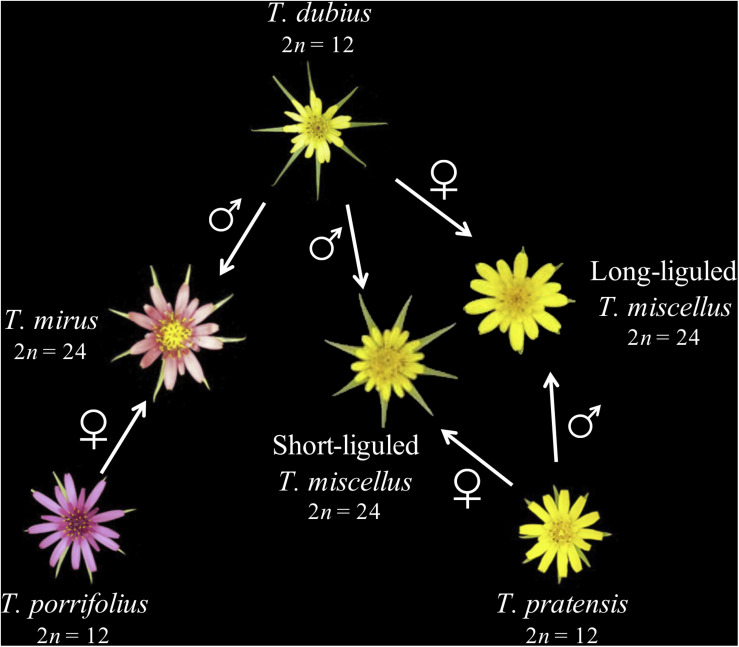
Parentage of two recently formed allotetraploid species of *Tragopogon*. Polyploid *T. miscellus* is derived from *T. dubius* and *T. pratensis* and has formed reciprocally: when *T. pratensis* is the maternal parent, *T. miscellus* is short-liguled; the long-liguled form has *T. dubius* as the maternal progenitor. The maternal and paternal parents of allopolyploid *T. mirus* are *T. porrifolius* and *T. dubius*, respectively.

*Tragopogon* (Asteraceae) is an outstanding natural system for studies of recent and recurrent allopolyploidy. Three diploid *Tragopogon* species (2*n* = 12), *T. dubius*, *T. pratensis*, and *T. porrifolius*, were introduced from Europe to the Pacific Northwest of North America in the early 1900s. Ownbey (1950) identified two new allotetraploids (2*n* = 24) native to the Palouse region of eastern Washington and adjacent Idaho, United States: *T. miscellus* and *T. mirus*. Both allotetraploids are only 90–100 years old (45–50 generations in these biennial plants) (Soltis et al., 2004). The parents of *T. miscellus* are *T. dubius* and *T. pratensis*, and those of *T. mirus* are *T. dubius* and *T. porrifolius* (Figure 1). Allotetraploids *T. miscellus* and *T. mirus* formed at least 21 and 11 times, respectively (Soltis et al., 2004; Symonds et al., 2010). Intriguingly, *T. miscellus* formed reciprocally with resultant distinct floral and inflorescence morphologies: those allotetraploids with *T. dubius* as the maternal parent have long ligules and open inflorescences, and those with *T. pratensis* as the maternal parent have short ligules and closed inflorescences (Ownbey, 1950; Ownbey and McCollum, 1953; Soltis and Soltis, 1989; Figure 1). Initial studies of the consequences of recurrent (including reciprocal) polyploidization in *T. miscellus* revealed similar patterns of stochastic homeolog loss and silencing among populations (including both short- and long-liguled forms) and a slight preferential loss of *T. dubius* homeologs (Tate et al., 2006, 2009; Buggs et al., 2009, 2011, 2012).

Allopolyploidy perturbs cytonuclear interactions, which may lead to biased homeolog expression and/or gene conversion toward the maternal parent in organelle-targeted nuclear genes (Gong et al., 2012, 2014; Sehrish et al., 2015; Sharbrough et al., 2017). Therefore, we hypothesize that organelle-targeted nuclear genes will display biased homeolog expression toward *T. dubius* and *T. pratensis* in long-liguled and short-liguled *T. miscellus*, respectively. In addition, unbalanced expression level dominance toward the maternal parent has been reported in many polyploids (reviewed in Yoo et al., 2014). If this hypothesis holds, the direction of unbalanced expression level dominance should be different between reciprocal origins of *T. miscellus*. Lastly, we attempt to address which gene(s) may be responsible for the different inflorescence morphology between reciprocally formed *T. miscellus*. For example, the roles of *CYC2* clade genes in determining flower type and controlling ligule growth have been demonstrated in many species of Asteraceae (reviewed in Elomaa et al., 2018). We can ask, do the *Tragopogon CYC2* orthologs express differentially between short- and long-liguled *T. miscellus*?

To dissect the gene expression changes following reciprocal polyploidization, inflorescence transcriptome dynamics of short- and long-liguled *T. miscellus* and their diploid parents were compared across ∼12,000 loci. We examined homeolog-specific expression in both ligule forms of *T. miscellus* by employing a robust Bayesian Poisson-Gamma model following methods in Boatwright et al. (2018). In addition, non-additive gene expression was analyzed in reciprocally formed *T. miscellus*, and differentially expressed loci were identified between short- and long-liguled *T. miscellus*. Previous studies of recurrent origins of *T. miscellus* employed only from 10 to 144 loci and mostly focused on leaf tissues (Tate et al., 2006, 2009; Buggs et al., 2009, 2011, 2012). By examining a much larger gene sample in the inflorescence, we can gain a more comprehensive understanding of the impact of reciprocal polyploidization on transcriptome dynamics in these naturally formed young polyploids.

## Materials and Methods

### Sample Collection and RNA Sequencing

*Tragopogon* plants were grown from field-collected seed in the Department of Biology greenhouse at the University of Florida, Gainesville, FL, United States. The following natural populations were analyzed in the current study (Table 1): *T. dubius* (Soltis and Soltis collection number 2613; Pullman, WA, United States), *T. dubius* (2886; Moscow, ID, United States), *T. pratensis* (2608; Moscow, ID, United States), short-liguled *T. miscellus* (2604; Moscow, ID, United States), and long-liguled *T. miscellus* (2605; Pullman, WA, United States). Because *T. pratensis* is extinct in Pullman, the paternal parent of long-liguled *T. miscellus* was represented by *T. pratensis* (2608) from Moscow in this study. Herbarium vouchers for all of these samples were deposited in the Florida Museum of Natural History Herbarium (FLAS).

**TABLE 1:** Populations of *Tragopogon* analyzed (vouchers are deposited at FLAS).

**Species**	**Soltis and Soltis collection number**	**Location**
*T. dubius*	2613	Pullman, WA
	2886	Moscow, ID
*T. pratensis*	2608	Moscow, ID
*T. miscellus* (short-liguled)	2604	Moscow, ID
*T. miscellus* (long-liguled)	2605	Pullman, WA

Fully opened inflorescences were collected from three individuals (one flower head per individual) of each sampled population and frozen in liquid nitrogen. RNA was extracted using a modified CTAB method (Jordon-Thaden et al., 2015). RNA-seq libraries were prepared using the NEBNext Ultra RNA Library Prep Kit for Illumina (New England Biolabs, Ipswich, MA, United States). Libraries from *T. miscellus* and the diploid parents were sequenced using Illumina NextSeq and HiSeq, respectively, to generate paired-end 150-bp reads. All sequencing was performed at the Interdisciplinary Center for Biotechnology Research (ICBR), University of Florida, Gainesville, FL, United States.

### Read Trimming

Approximately 135, 121, and 124 million raw paired-end reads were obtained from *T. dubius* (2613; Pullman), *T. dubius* (2886; Moscow), and *T. pratensis* (2608; Moscow), respectively (Supplementary Table S1). For *T. miscellus*, approximately 79 and 82 million raw paired-end reads were obtained from the short- and long-liguled form, respectively (Supplementary Table S1). Sequencing adaptors were removed using *Cutadapt* (version 2.1) (Martin, 2011). *Trimmomatic* (version 0.36) was used to remove low-quality bases (Bolger et al., 2014). Then, *sortmerna* (version 2.1) was used to remove rRNA sequences (Kopylova et al., 2012); 18S (accession number KT179662.1) and 26S (AF036493.1) ribosomal RNA genes from *T. dubius* were used as references.

### Transcript Assembly and Redundancy Removal

*Trinity* (version r20180213-2.6.5) was used to assemble high-quality, rRNA-free reads from the diploid parental species (Haas et al., 2013). Normalized reads (maximum and minimum coverages are 50 and 2, respectively) were used for *de novo* transcriptome assembly with default parameters. The genotype concordance between the two *T. dubius* populations (Pullman and Moscow) was calculated by identifying sequence variation (SNP and indel) using *Picard* GenotypeConcordance^1^ (Supplementary Table S2). Because of the high genotype concordance value, reads from the two populations of *T. dubius* were combined for the *T. dubius* transcript assembly (Supplementary Table S2). The quality of the assemblies was assessed by performing *BUSCO* analysis (Simão et al., 2015) and calculating ExN50 statistics (N50 value limited to the top highly expressed transcripts which account for x% of the total expression). In addition, by mapping reads back to assembled transcripts, the read composition of the assembly was assessed. To reduce assembly redundancy, *Lace* (Davidson et al., 2017) was used to generate SuperTranscripts, which comprise unique and common sequence regions from all isoforms derived from a single gene^2^.

### Ortholog Calling Between Diploid Parents

Reciprocal best-hit orthologs were identified between SuperTranscripts of *T. dubius* and *T. pratensis* following Boatwright et al. (2018). In addition, *OrthoFinder* (version 2.3.3) was used to identify single-copy orthogroups between the two diploid species using peptide sequences predicted by *TransDecoder* (version 5.5.0) (Haas et al., 2013; Emms and Kelly, 2015). By comparing results from the above two approaches, shared orthologous pairs with high similarity (*E*-value ≤ 1e-10, identity ≥ 80%, alignment length ≥ 200 bp; BLAST results using SuperTranscripts from *T. pratensis* as query and those from *T. dubius* as database) were isolated and used to examine gene expression patterns in downstream analyses.

### Homeolog-Specific Expression Analysis

**FIGURE 2:**
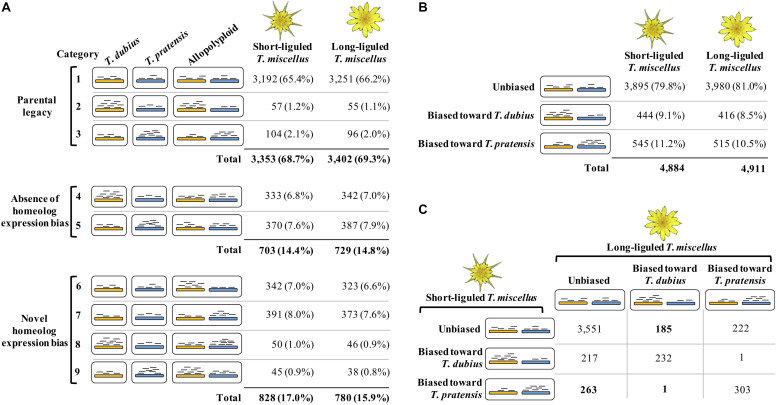
Homeolog-specific expression analysis in short- and long-liguled *T. miscellus*. The orange and blue bars represent homeologs derived from *T. dubius* and *T. pratensis*, respectively. Black short lines indicate reads that are mapped to the reference — the abundance of reads indicates the expression level. **(A)** Effects of parental gene expression on homeolog-specific expression. Parental legacy (categories 1–3) indicates that the expression level of a gene in the diploid parents is vertically transmitted to the polyploid. For example, in category 2, a gene shows higher expression in *T. dubius* than in *T. pratensis*; in the polyploid, the gene has biased homeolog expression toward *T. dubius*. Homeolog expression bias can be absent in the polyploid (categories 4 and 5). For example, although a gene is differentially expressed between the two parents, in the allopolyploid, the expression levels of the two homeologs are equivalent. Novel homeolog expression bias (categories 6–9) is biased homeolog expression toward one parent that does not show higher expression than the other parent. For example, in category 6, a gene is not differentially expressed between the two diploid parents, but biased homeolog expression toward *T. dubius* is observed in the polyploid. **(B)** The number and proportion of loci showing unbiased and biased homeolog expression in short- and long-liguled *T. miscellus*. **(C)** Comparison of the homeolog-specific expression profiles between short- and long-liguled *T. miscellus*. The number of loci showing “lineage-specific biased homeolog expression toward the maternal parent” is displayed in bold.

The Poisson-Gamma model (León-Novelo et al., 2014) was used to assess homeolog-specific expression in the polyploids following Boatwright et al. (2018). Boatwright et al. (2018) emphasized the importance of assessing read mapping bias prior to performing homeolog-specific expression analysis. Briefly, diploid read alignments identified orthologous pairs showing mapping bias, i.e., the diploid reads from one parent were predominantly mapped to the reference from the other parent. Homeolog-specific expression was then examined in the remaining orthologous pairs that did not exhibit biased mapping. In the polyploids, each orthologous pair was classified as one of the following three possible categories: unbiased homeolog expression, biased homeolog expression toward *T. dubius*, and biased homeolog expression toward *T. pratensis* (Figure 2B). In addition, we examined the effect of parental gene expression level on the relative homeolog expression in the allopolyploid, which included three types of expression patterns: parental legacy (Box 1 and Figure 2A, categories 1–3), absence of homeolog expression bias (Box 1 and Figure 2A, categories 4 and 5), and novel homeolog expression bias (Box 1 and Figure 2A, categories 6–9; Yoo et al., 2013, 2014).

### Differential Gene Expression Analysis

Reads mapped to both of the ‘common orthologous regions’ between orthologous pairs were evaluated for differential gene expression (per Boatwright et al., 2018). Differential gene expression was analyzed using *DESeq2* (version 1.24.0) with the negative binomial generalized linear model (Love et al., 2014). Read count normalization in *DESeq2* takes both sequencing depth and RNA composition into consideration. For quality control, at the sample level, principal component analysis (PCA) and hierarchical clustering were performed, and any sample outliers were identified; at the gene level, those loci with zero total read counts, low mean-normalized counts, and extreme count outliers were removed from further analysis. If the adjusted *P*-value was below 0.05 [i.e., false discovery rate (FDR) is less than 5% (Benjamini and Hochberg, 1995)], the locus (gene) was considered to be differentially expressed. Gene expression levels were compared among *T. dubius*, *T. pratensis*, and short- and long-liguled *T. miscellus*. In addition, differential gene expression was assessed between the polyploid and the mid-parent value (MPV) of its diploid parents.

### Analyses of Expression Level Dominance and Transgressive Expression

Differentially expressed loci between the polyploid and its diploid parents were parsed into various expression patterns (including additive expression, expression level dominance, and transgressive expression) according to Rapp et al. (2009) (Figure 4A). Briefly, if a gene was differentially expressed between the two diploids, additive expression indicates that the expression level in the polyploid is higher than one diploid parent but lower than the other one; a locus was classified as demonstrating expression level dominance when the expression level in the polyploid resembles that of one of the two parents; if the gene expression level in the polyploid was higher or lower than in both diploid parents, the locus was considered to show transgressive gene expression (Box 1). Following Yoo et al. (2013, 2014), in our study, non-additive expression includes both expression level dominance and transgressive expression (Figure 4A).

### *Trinotate* Annotation

*Trinotate* (version 3.0.1) was used to annotate the SuperTranscripts and predicted protein sequences (Bryant et al., 2017). BLAST similarities were captured using BLASTx and BLASTp from the UniProt protein database (release 2019_06) (UniProt Consortium, 2015); based on the Pfam database (release 32.0) (Finn et al., 2016), *HMMER* (version 3.2.1) was used to identify protein domains (Zhang and Wood, 2003); signal peptides were predicted by *SignalP* (version 5.0b) (Petersen et al., 2011); transmembrane regions and rRNA transcripts were identified by running *tmHMM* (version 2.0c) (Krogh et al., 2001) and *RNAMMER* (version 1.2) (Lagesen et al., 2007), respectively. All annotation results were loaded into a *Trinotate* SQLite Database (*E*-value threshold: 1e-5). Gene ontology (GO) assignments were obtained from UniProt and Pfam databases. Following Boatwright et al. (2018), the *GOseq* pipeline included in *Trinity* (version r20180213-2.6.5) was used for GO enrichment analysis by using GO terms derived from annotation of *T. dubius* assemblies (FDR < 0.05) (Young et al., 2010). The background gene set in the GO enrichment analysis included those loci used in assessing differential gene expression or homeolog-specific expression.

### Data Availability

The raw sequence reads have been uploaded to the BioProject database from NCBI (BioProject ID: PRJNA633300). All scripts used in data analysis, the assemblies, and annotations are available at https://github.com/GatorShan/Tragopogon-Inflorescence-RNA-seq-Analysis.

## Results

### *Trinity* Assembly and Redundancy Removal

After quality control, approximately 93 and 81% of reads remained in the diploid and polyploid samples, respectively (Supplementary Table S1). Using *de novo* assembly, in *T. dubius*, 126,278 *Trinity* genes and 302,750 *Trinity* transcripts were assembled with an N50 of 1,583 bp (based on all transcripts); GC percentage was 38.7%. In *T. pratensis*, the numbers of *Trinity* genes and *Trinity* transcripts were 99,228 and 239,956, respectively; GC percentage was 39.0%, and N50 was 1,482 bp (based on all transcripts).

*BUSCO* analysis was performed to assess completeness of the transcript assembly. Using 1,375 conserved single-copy orthologs from *Lactuca sativa* (Asteraceae) (a close relative of *Tragopogon*, in the same tribe; database: embryophyta_odb10) as the reference, 1,311 (95.3%) and 1,272 (92.5%) complete putative single-copy orthologs were identified in the *T. dubius* and *T. pratensis de novo* assemblies, respectively. In addition, when aligning RNA-seq reads back to *Trinity* assemblies, the overall alignment rate was 96.8% in both *T. dubius* and *T. pratensis*. Lastly, considered as a more appropriate criterion for transcriptome assembly evaluation than the N50 value, E90N50 statistics were computed by including the most highly expressed transcripts representing 90% of the total expression (Geniza and Jaiswal, 2017). E90N50 values were 1,802 and 1,663 bp for *T. dubius* and *T. pratensis de novo* assemblies, respectively. In summary, *de novo* assembly for the two diploid parents provided high-quality references for downstream gene expression analysis.

*Lace* was then used to remove isoform redundancy from the *Trinity de novo* assemblies: isoforms derived from a single gene were concatenated to create a SuperTranscript. For SuperTranscripts from *T. dubius*, the N50 value was 1,904 bp and the mean contig length was 921.8 bp. In *T. pratensis*, the N50 value and the average length of the SuperTranscripts were 1,954 and 981.7 bp, respectively. SuperTranscripts largely reduced redundancy of *Trinity* assemblies and were used to identify putative orthologs between the two diploid parents in the step noted below.

### Ortholog Identification in *T. dubius* and *T. pratensis*

Using SuperTranscripts reconstructed from the previous step, 42,595 reciprocal best-hit orthologs were found between the two diploid parents following Boatwright et al. (2018). In addition, *OrthoFinder* identified 18,341 single-copy orthogroups (one protein sequence per species) shared between *T. dubius* and *T. pratensis*. Of the 12,900 shared loci between reciprocal best-hit and *OrthoFinder* results, 11,863 orthologous pairs with high confidence (*E*-value ≤ 1e-10, identify ≥ 0.8, and alignment length ≥ 200 bp) were isolated for downstream gene expression analysis.

### Homeolog-Specific Expression in *T. miscellus*

Of 11,863 orthologous pairs identified in the two diploid parents, 5,400 loci showed unbiased mapping while aligning diploid reads to the SuperTranscripts. If reads from *T. dubius* mapped preferentially to the *T. pratensis* SuperTranscript, the orthologous pair was considered as showing mapping bias and was removed from the homeolog-specific expression analysis. Of the 5,400 unbiased orthologous pairs, 4,884 and 4,911 loci were examined in short- and long-liguled *T. miscellus*, respectively, along with their diploid parental populations. Three possible expression patterns are expected to be observed in polyploids: parental legacy, absence of homeolog expression bias, and novel homeolog expression bias (Box 1; Figure 2A).

The majority of orthologous pairs exhibited parental legacy in both forms of *T. miscellus* (68.7 and 69.3% in the short- and long-liguled forms, respectively) (Figure 2A, categories 1–3). For example, in short-liguled *T. miscellus*, the loci showing parental legacy included: (1) orthologous pairs that were not differentially expressed between *T. dubius* and *T. pratensis* and showed unbiased homeolog expression in the polyploid (65.4%); and (2) loci having biased homeolog expression toward the parent showing higher expression than that of the other parental species (3.3%) (Figure 2A). Following polyploidization, 14.4 and 14.8% of loci showed an absence of homeolog expression bias in short- and long-liguled *T. miscellus*, respectively (Figure 2A, categories 4 and 5). In addition, 17.0% of loci in short-liguled *T. miscellus* gained novel biased homeolog expression; in the long-liguled form, 15.9% of loci showed novel bias (Figure 2A, categories 6–9). Lastly, 81.9% of the loci examined (3,921 of 4,789) fell into the same category for both short- and long-liguled *T. miscellus* (Supplementary Figure S1).

Overall, 79.8 and 81.0% of orthologous pairs exhibited unbiased homeolog expression in short- and long-liguled *T. miscellus*, respectively (Figure 2B). In the short-liguled form, 444 (9.1%) and 545 (11.2%) loci showed biased homeolog expression toward *T. dubius* and *T. pratensis*, respectively (Figure 2B). In long-liguled *T. miscellus*, 416 (8.5%) loci displayed biased homeolog expression toward *T. dubius*, and the number of loci showing biased homeolog expression toward *T. pratensis* was 515 (10.5%) (Figure 2B). In both short- and long-liguled *T. miscellus*, unbalanced homeolog expression bias toward *T. pratensis* was found to be significant (*P*-value equals 1.3e-3 and 1.2e-3 in short- and long-liguled *T. miscellus*, respectively; chi-square goodness-of-fit test).

We then asked whether the same orthologous pairs showed consistent homeolog-specific expression patterns between short- and long-liguled *T. miscellus*. Of the 4,975 loci we examined, 4,086 orthologous pairs (82.1%) showed the same homeolog-specific expression profiles between the short- and long-liguled forms (Figure 2C). For example, for 232 loci, both short- and long-liguled *T. miscellus* had biased homeolog expression toward *T. dubius* (Figure 2C). When the homeolog-specific expression profiles differed between reciprocally formed *T. miscellus*, in most cases, orthologous pairs showing biased homeolog expression in one form (either long- or short-liguled) showed unbiased homeolog expression in the other form. In long-liguled *T. miscellus*, there were 185 and 222 loci that showed biased homeolog expression toward *T. dubius* and *T. pratensis*, respectively, in contrast, all of these loci exhibited unbiased homeolog expression in short-liguled *T. miscellus* (Figure 2C). In very rare cases (only 2 of 4,975 loci), the direction of homeolog expression bias was altered between short- and long-liguled forms (e.g., bias toward *T. dubius* in long-liguled *T. miscellus*, but toward *T. pratensis* in the short-liguled form) (Figure 2C).

### Differential Gene Expression Between *T. dubius* and *T. pratensis*

**FIGURE 3:**
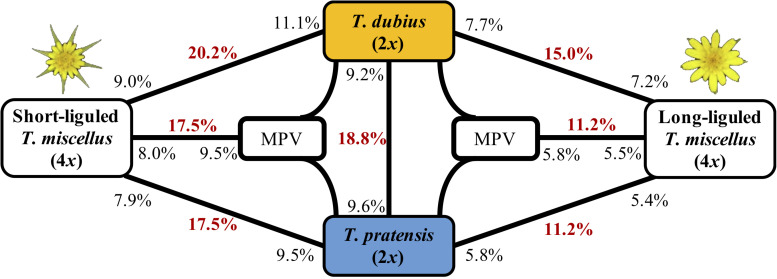
Differentially expressed genes between *T. dubius*, *T. pratensis*, and reciprocally formed *T. miscellus*. The number in bold red indicates the fraction of genes that are differentially expressed in each contrast. The number in black indicates the proportion of up-regulated genes, and the placement of the number indicates the direction of up-regulation. For example, 20.2% of loci were differentially expressed between *T. dubius* and short-liguled *T. miscellus*: 11.1% of the loci examined showed higher expression in *T. dubius* than in short-liguled *T. miscellus*; 9.0% of loci were more highly expressed in short-liguled *T. miscellus* than in *T. dubius*. MPV = mid-parent value.

Principal component analysis and hierarchical clustering analysis indicated that all samples from the same diploid species clustered together (Supplementary Figure S2). In addition, the genotype concordance analysis between the Pullman and Moscow populations of *T. dubius* showed a high sequence similarity (Supplementary Table S2), and only one locus showed differential gene expression between the two populations of *T. dubius* (Supplementary Figure S3). Therefore, data from all *T. dubius* individuals (from Pullman and Moscow) were combined for differential gene expression analysis. After quality control, 10,746 orthologous pairs remained. Between the two diploid species, 2,017 (18.8%) loci showed differential gene expression, and 987 (9.2%) and 1,030 (9.6%) loci were highly expressed in *T. dubius* and *T. pratensis*, respectively (Figure 3). We did not find any enriched GO term in the differentially expressed loci between *T. dubius* and *T. pratensis*.

### Differential Gene Expression Between Polyploids and Their Diploid Parents

Based on the hierarchical analysis, one individual of long-liguled *T. miscellus* (2605-9) did not cluster with the other two individuals from the same population (Supplementary Figure S4). In addition, the RNA integrity number (RIN) of individual 2605-9 was considerably lower than all other polyploid samples in this study (Supplementary Table S3). Because transcript quantification was likely affected by RNA integrity (Romero et al., 2014), this individual of long-liguled *T. miscellus* (2605-9) was removed in the differential gene expression analysis of this study. In terms of short-liguled *T. miscellus*, PCA and hierarchical analysis showed that all three replicates clustered together (Supplementary Figure S4).

The proportions of differentially expressed orthologous pairs between diploid parents and the two forms of *T. miscellus* are shown in Figure 3. Differential expression was found in 20.2% (2,128) of the loci examined between short-liguled *T. miscellus* and *T. dubius*; 17.5% (1,725) of orthologous pairs were differentially expressed between short-liguled *T. miscellus* and *T. pratensis* (Figure 3). In long-liguled *T. miscellus*, 15.0% (1,481) and 11.2% (1,103) of loci showed differential expression relative to *T. dubius* and *T. pratensis*, respectively (Figure 3).

Expression levels of orthologous pairs in polyploids were also compared with the mid-parent value (MPV) of the two diploid progenitors: 17.5% (1,768) and 11.2% (1,007) of loci were differentially expressed between the polyploid and the MPV in short- and long-liguled *T. miscellus*, respectively (Figure 3). In short-liguled *T. miscellus*, 8.0% (804) of loci were up-regulated relative to MPV, and 9.5% (964) of loci were down-regulated; in long-liguled *T. miscellus*, 5.5% (492) and 5.8% (515) of loci were up- and down-regulated compared to MPV, respectively (Figure 3).

### Expression Level Dominance and Transgressive Expression in *T. miscellus*

**FIGURE 4:**
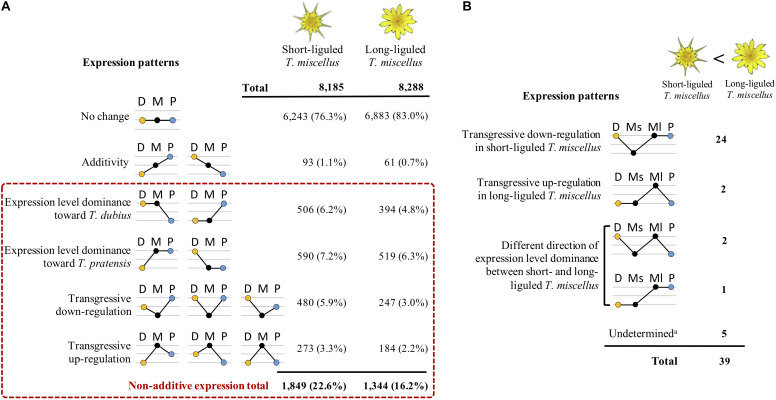
Differential expression patterns in reciprocally formed allopolyploid *T. miscellus* relative to their diploid parents. **(A)** Comparison of the number of loci belonging to various expression patterns between short- and long-liguled *T. miscellus*. Non-additive expression includes expression level dominance and transgressive expression. D = *T. dubius*, M = *T. miscellus*, P = *T. pratensis*. **(B)** The effect of non-additive expression on the 39 loci showing higher expression in long-liguled *T. miscellus* than in the short-liguled form. Ms = short-liguled *T. miscellus*, Ml = long-liguled *T. miscellus*. a: this includes loci that display differential expression between the two forms of *T. miscellus* and yet show equivalent expression between the polyploid and the diploid progenitors when the expression level of each form of *T. miscellus* is individually compared with its diploid parents.

By comparing the gene expression levels in the polyploid *T. miscellus* and its diploid parents, each locus was parsed into one of the expression patterns as shown in Figure 4A. In short- and long-liguled *T. miscellus*, 8,185 and 8,288 loci were examined, respectively. In the short-liguled form, the expression levels of 76.3% of the loci were not changed between the polyploid and its diploid parents; in the long-liguled form, 83.0% of the loci examined showed consistent expression between the polyploid and the diploid species (Figure 4A; expression pattern: no change). In addition, 1.1% of loci were additively expressed in short-liguled *T. miscellus*; in the long-liguled form, 0.7% of loci displayed additive expression (Figure 4A; expression pattern: additivity).

Non-additive expression, including expression level dominance and transgressive expression, was identified in 22.6 and 16.2% of the loci examined in short- and long-liguled *T. miscellus*, respectively (Figure 4A). Fisher’s exact test indicated that the proportions of non-additive loci were significantly different between the short- and long-liguled forms (*P*-value < 2.2e-16). In the short-liguled form, expression level dominance was unbalanced between the two subgenomes (*P*-value = 1.1e-2; chi-square goodness-of-fit test): 6.2 and 7.2% of loci showed expression level dominance toward *T. dubius* and *T. pratensis*, respectively (Figure 4A). In addition, in short-liguled *T. miscellus*, 5.9% of loci were transgressively down-regulated, and 3.3% of loci were transgressively up-regulated (Figure 4A). In the long-liguled form, expression level dominance toward *T. dubius* and *T. pratensis* was found in 4.8 and 6.3% of loci, respectively; a chi-square goodness-of-fit test showed that the expression level dominance was unbalanced toward *T. pratensis* (*P*-value = 3.5e-5) (Figure 4A). Additionally, 3.0 and 2.2% of loci were transgressively down- and up-regulated in long-liguled *T. miscellus*, respectively (Figure 4A).

Gene ontology enrichment analysis was performed using transgressively expressed loci in the polyploids. In short-liguled *T. miscellus*, the transgressively down-regulated loci showed enriched GO terms (biological process) in pectin catabolic process and cell wall modification (Supplementary Table S4); for transgressively up-regulated loci, no enriched GO term was found. In long-liguled *T. miscellus*, we did not find any overrepresented GO term in the transgressively expressed loci.

### Differential Gene Expression Between Reciprocally Formed *T. miscellus*

The transcriptomes of short- and long-liguled *T. miscellus* were compared, and 41 differentially expressed loci were identified. Of these loci, 39 orthologous pairs showed significantly higher expression in the long-liguled form than the short-liguled form; GO analysis indicated overrepresentation of genes involved in two biological processes: pectin catabolic process and cell wall modification (Supplementary Table S5). For the two genes that were significantly highly expressed in short-liguled *T. miscellus*, no GO term was enriched.

In addition, we analyzed the relationship of non-additive expression to differential gene expression in the reciprocal origins of *T. miscellus*. Of the 39 loci showing higher expression in long-liguled *T. miscellus* than the short-liguled form, the majority of loci (24 of 39) were transgressively down-regulated in short-liguled *T. miscellus* (Figure 4B). That is, the expression levels of these 24 loci were not changed between long-liguled *T. miscellus* and its diploid parents, and because these loci showed transgressive down-regulation in the short-liguled form, the expression levels of the 24 loci were higher in long-liguled *T. miscellus* relative to the short-liguled form.

We also assessed the relationship of homeolog-specific expression to differential gene expression. Homeolog-specific expression was examined in 24 of the 39 loci showing higher expression in long-liguled *T. miscellus* than in the short-liguled form. The majority of these loci (15 of 24; 62.5%) displayed unbiased homeolog expression in both short- and long-liguled *T. miscellus* (Supplementary Figure S5). Considering the two loci showing higher expression in the short-liguled form relative to the long-liguled form, homeolog-specific expression was examined in one locus which also showed unbiased homeolog expression in both forms. Therefore, the up-regulated gene expression observed in one ligule form was mainly due to the increased expression levels of both homeologs.

## Discussion

### Homeolog-Specific Expression in Polyploids

For each locus in the polyploid, homeolog-specific expression examines the relative contribution of homeologs derived from different diploid progenitors to the total gene expression. Biased homeolog expression might result from *cis*- and *trans*-regulatory changes (Wang et al., 2006a; Shi et al., 2012; Hu and Wendel, 2019) and/or DNA methylation dynamics: epigenetically silenced TEs can repress the expression of nearby homeologs, and variation in TE abundance and distribution between subgenomes may lead to homeolog expression bias (reviewed in Wendel et al., 2018). In the long run, homeologs with lower expression can be preferentially lost, which ultimately promotes biased fractionation (reviewed in Wendel et al., 2018). In addition, Yang et al. (2016) indicated that genes showing homeolog expression bias had more selection potential than genes displaying unbiased homeolog expression in polyploid *Brassica*, a phenomenon with potential agricultural applications for accelerating the breeding process.

Homeolog-specific expression has been studied in both natural and synthetic systems, including *Arabidopsis* (Wang et al., 2006a; Chang et al., 2010), *Brachypodium* (Takahagi et al., 2018), *Brassica* (Yang et al., 2016), *Coffea* (Combes et al., 2013), *Glycine* (Coate et al., 2014), *Gossypium* (Yoo et al., 2013; Rambani et al., 2014), *Mimulus* (Edger et al., 2017), *Polypodium* (Sigel et al., 2019), *Tragopogon* (Buggs et al., 2010; Boatwright et al., 2018), *Triticum* (Nomura et al., 2005), and *Zea* (Schnable et al., 2011).

Buggs et al. (2010) studied homeolog-specific expression in leaf tissue of short-liguled *T. miscellus* (2671; Oakesdale, WA, United States) and found that 69% of the loci examined showed equal expression of both homeologs. Also using leaf tissue, Boatwright et al. (2018) examined homeolog-specific expression of short-liguled *T. miscellus* from another population (2894-2; Garfield, WA, United States); the long-liguled form was not examined. Approximately 48% of loci showed unbiased homeolog expression, and 26.2 and 25.4% of orthologous pairs had biased homeolog expression toward *T. dubius* and *T. pratensis*, respectively (Boatwright et al., 2018). We found that, in the inflorescence of both short- and long-liguled *T. miscellus*, the majority of loci (∼80%) showed unbiased homeolog expression. In the short-liguled form, 9.1 and 11.2% of loci exhibited biased homeolog expression toward *T. dubius* and *T. pratensis*, respectively (Figure 2B).

As shown above, in short-liguled *T. miscellus*, the proportions of loci showing unbiased homeolog expression were divergent among studies. The difference may result from the method employed to assess homeolog-specific expression. In Buggs et al. (2010), the origin of a *T. miscellus* read was determined by using homeolog-specific single nucleotide polymorphism (SNP) markers between the diploid parents; instead, in Boatwright et al. (2018) and the current study, the analysis of homeolog-specific expression was based on a robust Bayesian Poisson-Gamma model for thousands of loci. In addition, different populations of short-liguled *T. miscellus* were analyzed in these studies, and divergent homeolog-specific expression profiles may be present at the population level. Lastly, Buggs et al. (2010) and Boatwright et al. (2018) examined homeolog-specific expression in leaf tissues, but the inflorescence transcriptome was analyzed in our study. The impact of tissue type on homeolog-specific expression is discussed below.

Homeolog-specific expression in allotetraploid *Gossypium hirsutum* has been examined using both leaf and petal transcriptomes (Yoo et al., 2013; Rambani et al., 2014): in cultivar “Maxxa,” 73.2 and 79.4% of the genes examined showed unbiased homeolog expression in leaf and petal tissues, respectively; in a wild accession “TX2094,” unbiased homeolog expression was found in 68.8% of genes in leaves, and 80.2% of the genes examined displayed equivalent expression of both homeologs in petals. In both leaf and petal tissues, biased homeolog expression was balanced (Yoo et al., 2013; Rambani et al., 2014). In allopolyploid *Brachypodium hybridum*, ∼60% of the genes showed unbiased homeolog expression in both leaf and root tissues (Takahagi et al., 2018). In summary, our results indicate that tissue type (leaf vs. inflorescence) may have an impact on relative homeolog expression.

Because *T. miscellus* is only 90–100 years old (45–50 generations) (Soltis et al., 2004), the extant diploid parental species are expected to have similar expression patterns compared to the exact ancestors of the polyploids. Therefore, the effects of parental gene expression profiles on relative homeolog expression in the polyploids can be rigorously examined in *Tragopogon*, which is unique among various well-studied polyploid systems for this reason (Soltis and Soltis, 2009). In short- and long-liguled *T. miscellus*, 68.7 and 69.3% of orthologous pairs showed parental legacy (Figure 2A), indicating divergence from parental patterns at the remaining loci. In both forms of *T. miscellus*, approximately 15 and 16% of the loci examined displayed an absence of homeolog expression bias and novel homeolog expression bias in polyploids, respectively (Figure 2A). The percentage of loci showing parental legacy in *Tragopogon* is comparable to the results from other polyploid systems: ∼63 and ∼65% of analyzed genes showed parental legacy in natural allopolyploid *Gossypium* (Yoo et al., 2013) and allopolyploid *Brachypodium* (Takahagi et al., 2018), respectively. The fact that only approximately two-thirds of all loci examined in *T. miscellus* showed parental legacy after fewer than 50 generations of evolution of the polyploid indicates that deviations from parental patterns arise quickly after polyploid formation. This process occurs so rapidly that a young polyploid species such as *T. miscellus* has already undergone shifts in gene expression characteristic of much older polyploid species.

### The Effect of Cytonuclear Interaction on Homeolog-Specific Expression

Cytonuclear incompatibility may occur following allopolyploidization: the nuclear genome is inherited from both parents, but the cytoplasmic genome is derived, typically, from the maternal parent (reviewed in Mogensen, 1996). Coordinated expression between cytoplasmic and nuclear genes is favored by selection following allopolyploidization. One intriguing potential compensatory mechanism is biased homeolog expression and/or gene conversion toward the maternal parent in organelle-targeted nuclear genes (Sharbrough et al., 2017). However, this hypothesis has only been tested with the *rbcS* gene (encodes the small subunit of Rubisco); asymmetric gene conversion biased toward the maternal copy has been reported (Gong et al., 2012, 2014).

Reciprocally formed *T. miscellus* provides an excellent system to examine the effect of cytonuclear interaction on homeolog-specific expression in allopolyploids. In this study, 17.9% of orthologous pairs (889 loci) showed different homeolog expression patterns between short- and long-liguled *T. miscellus* (Figure 2C). We isolated 449 loci with lineage-specific biased homeolog expression toward the maternal parent (Figure 2C, loci shown in bold). For example, this set of orthologous pairs included 185 loci showing biased homeolog expression toward *T. dubius* in long-liguled *T. miscellus* (of which *T. dubius* was the maternal parent), but unbiased homeolog expression in the short-liguled form. We hypothesized that if cytonuclear incompatibility resulted in biased homeolog expression toward the maternal parent, organelle-targeted nuclear genes should be included in these 449 loci and biological processes relevant to cytonuclear interactions (e.g., regulation of photosynthesis and oxidative phosphorylation) should be overrepresented in the GO analysis. However, no enriched GO term was found in these loci. In addition, genes with a mitochondrial/chloroplast transit peptide were not enriched in the 449 loci (Supplementary Figure S6). Consistent with our results, Sehrish et al. (2015) revealed that for the *rbcS* gene, a low proportion (16%) of naturally occurring *T. miscellus* individuals exhibited biased homeolog expression toward the maternal parent, and all of the synthetic *T. miscellus* individuals examined showed unbiased homeolog expression. Therefore, cytonuclear coordination may not be established immediately following polyploidization (Sehrish et al., 2015), and the number of loci showing biased homeolog expression toward the maternal parent is still low in the very young (90–100 years old) *T. miscellus*, which is, therefore, not captured by the GO analysis.

### Non-additive Gene Expression in Polyploids

Non-additive gene expression, including expression level dominance and transgressive expression, is prevalent in diverse polyploid systems (reviewed in Yoo et al., 2014). The proportion of loci displaying non-additive expression varied significantly among different species, tissues, and environmental conditions. In allotetraploid *Arabidopsis suecica*, ∼5-6% of loci displayed non-additive expression (Wang et al., 2006b). In resynthesized allopolyploid wheat, non-additive expression was found in ∼19% of the loci examined (Akhunova et al., 2010). In *Gossypium hirsutum* cultivar “Maxxa,” ∼29 and 61% of loci displayed non-additive expression in leaf and petal tissues, respectively (Yoo et al., 2013, 2014; Rambani et al., 2014). In allotetraploid *Coffea arabica*, ∼43 and 60% of loci showed non-additive expression under low and high temperatures, respectively (Bardil et al., 2011).

In our study, 22.6 and 16.2% of loci were non-additively expressed in short- and long-liguled *T. miscellus*, respectively (Figure 4A). These results mirrored those of the differential gene expression analysis between the polyploids and diploid progenitors: relative to the short-liguled form, fewer differentially expressed genes were found between the long-liguled form and either of the diploid parents (Figure 3).

In many polyploid species, unbalanced expression level dominance toward the maternal parent has been observed (reviewed in Yoo et al., 2014). For example, in leaf tissue of *Gossypium hirsutum* cultivar “Maxxa,” 9.3 and 4.0% of loci showed expression level dominance toward the maternal parent (the A-genome) and paternal parent (the D-genome), respectively (Yoo et al., 2014). Similarly, in allopolyploid *Tragopogon mirus*, 8.5 and 7.8% of loci displayed expression level dominance toward *T. porrifolius* (the maternal parent) and *T. dubius* (the paternal parent), respectively (Yoo et al., unpublished).

The maternal influence on non-additive expression may result from: (1) the maternal inheritance of the cytoplasmic genome; and (2) cytonuclear incompatibilities (reviewed in Yoo et al., 2014; as described above in “The effect of cytonuclear interaction on homeolog-specific expression”). However, in the current study, irrespective of the cross direction, unbalanced expression level dominance toward *T. pratensis* was observed (Figure 4A). Consistent with these results, cytonuclear incompatibility did not result in biased homeolog expression toward the maternal parent among organelle-targeted nuclear genes in *T. miscellus*. In allotetraploid *Polypodium hesperium*, unbalanced expression level dominance toward diploid *P. amorphum* was found in both reciprocal origins of the polyploid (Sigel et al., 2019). In addition, unbalanced expression level dominance toward the paternal parent was also found in polyploid *Coffea* (Bardil et al., 2011) and *Gossypium* (Rapp et al., 2009). More research is clearly needed on additional polyploid systems having reciprocal formations.

Interestingly, the transgressively down-regulated loci in short-liguled *T. miscellus* showed enriched GO terms in pectin catabolic process and cell wall modification (Supplementary Table S4); the exact two GO terms were overrepresented among loci showing higher expression in long-liguled *T. miscellus* than the short-liguled form (Supplementary Table S5) (the potential impact of these loci on morphology is discussed below). Consistent with these results, 24 of the 39 loci showing higher expression in long-liguled *T. miscellus* relative to the short-liguled form were transgressively down-regulated in short-liguled *T. miscellus* (Figure 4B). Therefore, the distinct non-additive expression patterns between reciprocal origins provide increased genetic diversity in the polyploids, which might lead to their success.

### Distinctive Inflorescence Morphology Between Reciprocally Formed *T. miscellus*

Asteraceae are well known for their unique inflorescence (i.e., capitulum or flower head) — a key morphological innovation that is associated with their evolutionary success (Broholm et al., 2014). The flower head can be either homogamous (i.e., comprising one floral type) or heterogamous (i.e., comprising both ray and disk flowers). *Tragopogon* has flower heads composed of ligulate flowers only.

Previous studies have shown the important role of genes from the *CYC2* clade of the *CYCLOIDEA/TEOSINTE BRANCHED1* (*CYC/TB1*) gene family in defining floral identity and controlling ligule growth in other Asteraceae, including *Gerbera*, *Helianthus*, *Senecio*, and *Chrysanthemum* (reviewed in Elomaa et al., 2018). In developing flower primordia of *Gerbera* and *Helianthus*, *CYC2* clade genes exhibit higher expression in ray flower primordia than in disk flower primordia (Broholm et al., 2008; Tähtiharju et al., 2012). Disrupted expression of *CYC2* genes affects ligule length in ray and/or disk flowers in a species-specific manner (reviewed in Elomaa et al., 2018). In addition, at later stages of inflorescence development (including the fully opened inflorescence), *CYC2* clade genes are highly expressed in floral reproductive organs (stamen, stigma, style, and ovary) in *Gerbera* and *Helianthus* (Tähtiharju et al., 2012). However, how *CYC2* clade genes function in Asteraceae species with homogamous flower heads, such as *Tragopogon*, is still unknown.

We identified six SuperTranscripts (from *T. dubius*) that were homologous to the 13 *T. dubuis CYC/TB1* clade genes identified by Liu (2018) in a genome assembly of *T. dubius*. The expression profiles of these SuperTranscripts were examined, and none of them were differentially expressed between short- and long-liguled *T. miscellus* (Supplementary Table S6). Therefore, *CYC/TB1* clade genes may not be a factor in ligule length in *Tragopogon*. However, to better assess the role of *CYC2* genes in controlling ligule length in *Tragopogon*, future studies should also examine inflorescence transcriptomes from very early developmental stages — in *Gerbera* and *Helianthus*, it is in primordia that *CYC2* genes have shown differential expression between floral types with different ligule length (Elomaa et al., 2018). If the ligule length difference between reciprocally formed *T. miscellus* results from differential expression of *CYC2* orthologs in floral primordia, the differential expression of *CYC2* clade genes might not be captured by transcriptomes from inflorescences at anthesis (the material available and examined in this study). In addition, *CYC2* genes are expressed in reproductive organs at later inflorescence developmental stages in *Gerbera* and *Helianthus* (Tähtiharju et al., 2012). Therefore, to examine the function of *CYC2* genes in determining ligule growth, organ-specific transcriptomic studies of ligulate flowers would also be useful in future studies of *Tragopogon*.

In our study, 39 differentially expressed orthologous pairs were identified with higher expression in long-liguled *T. miscellus* compared to the short-liguled form. We are especially interested in the two significantly overrepresented GO terms of these loci: pectin catabolic process and cell wall modification (Supplementary Table S5). Pectin is a prominent component of the primary cell wall and the middle lamella. Numerous studies have indicated the important role of pectin modification in the regulation of cell wall extensibility, which has an impact on plant growth (reviewed in Palin and Geitmann, 2012; Wolf and Greiner, 2012). On the one hand, pectinesterase catalyzes the demethylesterification of pectin. In shoot apical meristems, pectin demethylesterification softens the cell wall and triggers primordium formation: overexpression of pectinesterase results in an increased number of primordia, and in turn, overexpression of pectinesterase inhibitor blocks primordium formation (Palin and Geitmann, 2012; Wolf and Greiner, 2012). In our study, pectinesterase activity is the most significantly overrepresented GO term in molecular function (FDR = 3.5e-3).

Another gene of interest encodes polygalacturonase. This enzyme cleaves demethylated pectin, which loosens the cell wall and enables cell expansion (Wolf and Greiner, 2012). Changes in polygalacturonase expression can perturb normal floral organ patterning in *Arabidopsis* (Xiao et al., 2014). A higher proportion of flowers had extra petals in both *PGX1* (*POLYGALACTURONASE INVOLVED IN EXPANSION1*) overexpression and mutant plants compared to that of the wild type counterparts (Xiao et al., 2014). In our study, pectin catabolic process is significantly enriched among differentially expressed loci showing higher expression in long-liguled *T. miscellus* than the short-liguled form (Supplementary Table S5).

Therefore, we speculate that the very few genes involved in pectin metabolism (especially genes controlling pectinesterase and polygalacturonase activity) may affect primordia formation and floral organ patterning in *Tragopogon*, and thereby contribute to the morphological differences in inflorescence structure between reciprocal formations of *T. miscellus* (short- vs. long-liguled forms) — they should be a focus of more study. In plants, major morphological changes may be governed by just a few genes. For example, Hilu (1983) proposed the role of single-gene mutation in major morphological shifts in plants. Similarly, Gottlieb (1984) argued that very few genes might be responsible for structure, shape, and orientation changes. Doebley and Stec (1991) found that five genomic regions (one region included the *tb1* gene) could explain the dramatic inflorescence morphology difference between maize and teosinte. In a recent study of the carnivorous plant *Utricularia gibba*, the ectopic expression of a single gene (*UgPHV1*) impeded trap formation (Whitewoods et al., 2020).

Our hypothesis requires further study — an effect of pectin metabolism on inflorescence development has not been reported in Asteraceae. With an established CRISPR/Cas9 system in *Tragopogon* (Shan et al., 2018, 2020), the functions of related genes (such as genes encoding pectinesterase and polygalacturonase) could be rigorously examined.

In addition, there are two other important directions for future studies. First, the very few differentially expressed loci between the two ligule forms identified in our study may be targeted by a single transcription factor (TF). To test this hypothesis, the promoter sequences of the differentially expressed genes can be analyzed to identify any TF binding site(s). Second, the effect of the cytoplasm and cytonuclear interaction on ligule growth should be examined. Ownbey and McCollum (1953) crossed the diploid species of *Tragopogon* and assessed the inflorescence phenotype of the F_1_ and F_2_ generations. They found that ligule development was inhibited in the F_1_ hybrids with the *T. pratensis* cytoplasm, and the subsequent F_2_ generation displayed remarkable variation in the degree of ligule inhibition (Ownbey and McCollum, 1953). Therefore, future studies could compare the genotype and expression level of organellar genes (from plastids and mitochondria) between *T. dubius* and *T. pratensis*, and analyze the gene expression networks between organellar and nuclear genes in both diploids and their polyploid derivative (*T. miscellus*). All of these efforts will help provide a better understanding of the molecular mechanisms underlying ligule growth in *Tragopogon*.

## Data Availability Statement

The datasets presented in this study can be found in online repositories. The names of the repository/repositories and accession number(s) can be found below: https://www.ncbi.nlm.nih.gov/bioproject/, BioProject ID: PRJNA633300.

## Author Contributions

DS, PS, and SS designed the research. SS, XL, and CF collected the materials. SS, XL, and AC extracted RNA and constructed the libraries. SS and JB analyzed the data. SS drafted the manuscript. DS, PS, JB, XL, and AC participated in the writing of the manuscript. All authors read and approved the final version of the manuscript.

## Conflict of Interest

The authors declare that the research was conducted in the absence of any commercial or financial relationships that could be construed as a potential conflict of interest.

## Footnotes

1 http://broadinstitute.github.io/picard

2 https://github.com/trinityrnaseq/trinityrnaseq/wiki/SuperTranscripts
