# Supplementary material for: Transcriptome Dynamics of the Inflorescence in Reciprocally Formed Allopolyploid Tragopogon miscellus (Asteraceae)
Source: Front Genet. 2020 Aug 6;11:888. doi: 10.3389/fgene.2020.00888 (PMC7423994; doi:10.3389/fgene.2020.00888)
Supplement: Supplementary file 1 [file Data_Sheet_1.docx]

**Supplementary Materials**

Supplementary Table S1. Summary of transcriptome data.

|  | Species | Individual | Raw reads  (paired-end) | Trimmed reads  (paired-end) | % of reads remained after trimming |
| --- | --- | --- | --- | --- | --- |
|  | *T. dubius* (2*x*) | 2613-11 | 37,469,422 | 34,596,249 | 92.3 |
|  |  | 2613-12 | 46,452,532 | 43,105,595 | 92.8 |
|  |  | 2613-41 | 51,370,932 | 47,740,672 | 92.9 |
|  |  | 2886-3 | 34,587,668 | 32,180,016 | 93.0 |
|  |  | 2886-5 | 47,788,858 | 44,623,798 | 93.4 |
|  |  | 2886-7 | 38,347,993 | 35,730,610 | 93.2 |
|  | *T. pratensis* (2*x*) | 2608-3 | 34,744,553 | 32,151,059 | 92.5 |
|  |  | 2608-21 | 60,543,855 | 55,913,388 | 92.4 |
|  |  | 2608-31 | 28,925,754 | 26,809,659 | 92.7 |
|  | *T. miscellus*  (short-liguled) (4*x*) | 2604-24 | 25,875,630 | 21,496,636 | 83.1 |
|  |  | 2604-43 | 25,583,468 | 21,043,277 | 82.3 |
|  |  | 2604-48 | 27,910,493 | 22,491,439 | 80.6 |
|  | *T. miscellus*  (long-liguled) (4*x*) | 2605-9 | 29,023,629 | 23,275,437 | 80.2 |
|  |  | 2605-24 | 27,362,115 | 21,933,039 | 80.2 |
|  |  | 2605-42 | 25,286,876 | 20,807,972 | 82.3 |

Supplementary Table S2. Genotype concordance analysis between *T. dubius* (2613; Pullman) and *T. dubius* (2886, Moscow).

| Variant type | Genotype concordance (a/b) | Number of times the two populations’ variant states match exactly (a) | All variant states combinations between the two populations (b) |
| --- | --- | --- | --- |
| SNP | 81.1% | 94,701 | 116,828 |
| Indel | 71.6% | 9,420 | 13,163 |

Note: *HISAT2* was used to map *T. dubius* trimmed reads to *T. dubius* draft reference genome (Kim et al., 2015; Liu, 2018); VCF files were generated by using *freebayes* (Garrison and Marth, 2012); genotype concordance analysis was performed by using *Picard* GenotypeConcordance (<http://broadinstitute.github.io/picard>).

Supplementary Table S3. RNA integrity number (RIN) of allotetraploid species of *Tragopogon*

| Species | Individual | RIN |
| --- | --- | --- |
| *T. miscellus*  (short-liguled) (4*x*) | 2604-24 | 9.1 |
|  | 2604-43 | 9.1 |
|  | 2604-48 | 8.8 |
| *T. miscellus*  (long-liguled) (4*x*) | 2605-9 | 2.5 |
|  | 2605-24 | 9.1 |
|  | 2605-42 | 8.9 |

Supplementary Table S4. Enriched GO terms in biological process ontology of orthologous pairs showing transgressive down-regulation in short-liguled *T. miscellus*.

| GO-ID | Term | False discovery rate (FDR) |
| --- | --- | --- |
| GO:0045490 | pectin catabolic process | 6.0e-5 |
| GO:0042545 | cell wall modification | 2.1e-2 |

Supplementary Table S5. Enriched GO terms in biological process ontology of orthologous pairs showing higher expression in long-liguled *T. miscellus* than in short-liguled *T. miscellus*.

| GO-ID | Term | False discovery rate (FDR) |
| --- | --- | --- |
| GO:0045490 | pectin catabolic process | 3.0e-5 |
| GO:0042545 | cell wall modification | 3.5e-3 |

Supplementary Table S6. Normalized reads count matrix of *T. dubius* SuperTranscripts that are homologous to *T. dubius* CYC/TB1 clade genes in short- and long-liguled *T. miscellus*.

| *T. dubius* SuperTranscripts | Read counts | | | | | |
| --- | --- | --- | --- | --- | --- | --- |
|  | Tml_1 | Tml_2 | Tml_3 | Tms_1 | Tms_2 | Tms_3 |
| Tdu_TRINITY_DN18324_c0_g4 | 38.3 | 20.7 | 26.6 | 16.6 | 33.0 | 25.4 |
| Tdu_TRINITY_DN18324_c0_g2 | 4.0 | 0 | 1.0 | 0.9 | 1.0 | 0 |
| Tdu_TRINITY_DN19548_c1_g2 | 0 | 4.1 | 3.1 | 0 | 0 | 2.6 |
| Tdu_TRINITY_DN9954_c0_g1 | 0 | 0 | 0 | 0 | 1.0 | 2.0 |
| Tdu_TRINITY_DN19040_c6_g3 | 5.3 | 0 | 3.1 | 1.8 | 7.2 | 2.0 |
| Tdu_TRINITY_DN25185_c2_g1 | 63.4 | 23.8 | 25.6 | 16.6 | 35.1 | 24.1 |

Note: Tdu = *T. dubius*; Tml = long-liguled *T. miscellus*; Tms = short-liguled *T. miscellus.* There are three replicates of short- and long-liguled *T. miscellus*.

Supplementary Figure S1. Comparison of the effects of parental gene expression on homeolog-specific expression between short- and long-liguled *T. miscellus*. The orange and blue bars represent homeologs derived from *T. dubius* and *T. pratensis*, respectively. Black short lines indicate reads that are mapped to the reference — the abundance of reads indicates the expression level.

Supplementary Figure S2. Principal component analysis (A) and hierarchical clustering heatmap (B) of samples from *T. dubius* and *T. pratensis*. Tdu = *T. dubius*; Tpr = *T. pratensis*. *T. dubius* and *T. pratensis* have six and three replicates, respectively.

Supplementary Figure S3. MA-plot between the Pullman and Moscow populations of *T. dubius*. The x-axis represents the average normalized counts for each gene across the two populations; the y-axis represents the log2 fold change of the normalized counts for each gene between the two populations. The red point (within the circle) indicates the differentially expressed gene (adjusted *P*-value was below 0.05). Points below the red line (y = 0) represent genes showing higher expression in the Moscow population than in the Pullman population.

Supplementary Figure S4. Principal component analysis (A) and hierarchical clustering heatmap (B) of samples from *T. dubius*, *T. pratensis*, and *T. miscellus*. Tdu = *T. dubius*; Tpr = *T. pratensis*; Tml = long-liguled *T. miscellus*; Tml_1 = individual 2605-9; Tms = short-liguled *T. miscellus*. There are three replicates of *T. pratensis*, short-liguled *T. miscellus*, and long-liguled *T. miscellus*; *T. dubius* has six replicates.

Supplementary Figure S5. Homeolog-specific expression profiles of loci showing higher expression in long-liguled *T. miscellus* than the short-liguled form.

Supplementary Figure S6. The proportions of organelle-targeted loci in the background gene set and the 449 loci with lineage-specific biased homeolog expression toward the maternal parent. *TargetP* (version 2.0) was used to predict the presence of mitochondrial/chloroplast transit peptide (Armenteros et al., 2019). The background gene set included 4,975 loci which were used for comparing homeolog-specific expression profiles between the two forms of *T. miscellus*. Fisher’s exact test indicated that the proportions of organelle-targeted loci were not significantly different between the 449 loci and the background gene set (*P*-value = 0.17).

**References**

Armenteros, J. J. A., Salvatore, M., Emanuelsson, O., Winther, O., von Heijne, G., Elofsson, A., et al. (2019). Detecting sequence signals in targeting peptides using deep learning. *Life Sci. Alliance* 2, 5. doi:10.26508/lsa.201900429.

Garrison, E., and Marth, G. (2012). Haplotype-based variant detection from short-read sequencing. *arXiv* [Preprint]. Available at: <https://arxiv.org/abs/1207.3907>.

Kim, D., Langmead, B., and Salzberg, S. L. (2015). HISAT: A fast spliced aligner with low memory requirements. *Nat. Methods* 12, 357–360. doi:10.1038/nmeth.3317.

Liu, X. (2018) Alternative splicing in basal angiosperms and *Tragopogon* (Asteraceae). [dissertation]. [Gainesville (FL)]: University of Florida
